# Age-Related Longitudinal Changes in Metabolic Energy Expenditure during Walking in Boys with Duchenne Muscular Dystrophy

**DOI:** 10.1371/journal.pone.0115200

**Published:** 2014-12-15

**Authors:** Merel-Anne Brehm, Jiska C. E. Kempen, Anneke J. van der Kooi, Imelda J. M. de Groot, Janneke C. van den Bergen, Jan J. G. M. Verschuuren, Erik H. Niks, Jaap Harlaar

**Affiliations:** 1 Department of Rehabilitation Medicine and MOVE Research Institute, VU University Medical Center, Amsterdam, The Netherlands; 2 Department of Rehabilitation, Academic Medical Center, University of Amsterdam, The Netherlands; 3 Department of Neurology, Academic Medical Center, University of Amsterdam, The Netherlands; 4 Department of Rehabilitation, Radboud University Medical Center Nijmegen, The Netherlands; 5 Department of Neurology, Leiden University Medical Center, Leiden, The Netherlands; Stem Cell Research Institute, Belgium

## Abstract

**Objective:**

The aim of this study was to evaluate age-related changes in metabolic walking energy expenditure in ambulant boys affected by Duchenne muscular dystrophy over a follow-up period of 12 months.

**Methods:**

At baseline (T1) and 12 months later (T2), metabolic walking energy expenditure was assessed during a 6-minute walk test at comfortable speed in 14 ambulant boys with Duchenne (age range: 6.0-12.5 years, mean 8.2). Outcome measures derived from the assessment included the 6-minute comfortable walking distance (m) and net-nondimensional energy cost relative to speed-matched control cost (SMC-EC, %). Statistical comparisons were made using a two-way repeated measures ANOVA (factors: time (T1 versus T2) and age (<8 years of age (yoa) versus ≥8 yoa)).

**Results:**

Over the course of the study, a significant decrease of -28m (−8.2%, p = 0.043) was noted in the walked distance at comfortable speed. Besides, SMC-EC increased with 4.4%, although this change was not significant (p = 0.452). Regarding age groups, boys below 8 yoa showed a smaller annual decrease in the walked distance (−15 m) compared to boys above 8 yoa (−37 m). SMC-EC increased with 10% in the older boys, while in the younger boys it decreased (−2.1%). The main effect of age group on walking distance and SMC-EC however was not significant (p>0.158), and also there were no interaction effects (p>0.248).

**Conclusions:**

The results of our small study suggest that the natural course of walking performance in ambulant boys with Duchenne is characterized by a decrease in comfortable walking distance and an increase in walking energy cost. The rate of energy cost seems to increase with age, while walking distance decreases, which is opposite from the trend in typically developing children.

## Introduction

Duchenne muscular dystrophy (DMD) is the most common neuromuscular disease of childhood, affecting 1 in 5000 newborn boys [1]. The disease is caused by mutations in the *DMD* gene that encodes the dystrophin protein [2]. Affected boys develop severe, progressive muscle weakness that leads to loss of ambulation during childhood. An important aim in the management of DMD is therefore to delay the age of onset of loss of ambulation [3], which subsequently promotes mobility-related activities of daily life.

Prior to the loss of ambulation, muscle weakness leads to poor walking endurance [4], [5] and a severely increased walking energy expenditure [5], which is a major cause of early fatigability during gait [6]. As being able to walk greater distances with less physical fatigue has a significant positive impact on social participation and quality of life [7], reducing walking energy expenditure should therefore be considered a key treatment goal during the ambulatory phase of DMD [3]. Understanding the natural course of walking energy expenditure in DMD patients will assist in determining the need and timing of treatment interventions for boys whose walking energy expenditure is high. Such understanding is especially relevant, given that loss of ambulation in DMD occurs against a background of normal growth and maturation. [8], [9] Based on longitudinal data describing age-related declines in walking distance in boys with DMD [10]–[14], a progressive increase in walking energy expenditure is the expected natural course. However, no information supporting this hypothesis yet exists for boys with DMD. The objective of this study was therefore to evaluate age-related changes in the metabolic walking energy expenditure in ambulant boys with DMD over a follow-up period of 1 year. Considering the age dependent biphasic pattern of evolution of walking performance reported in previous studies [13], [14], both the effects of time and age on walking energy expenditure were evaluated.

## Methods

### Participants

The boys included in this study were recruited from the All Against Duchenne in the Netherlands (ALADIN) network. The inclusion criteria were: a confirmed DNA diagnosis of DMD, aged at least 6 years, and capable of walking more than 150m with or without the use of a walking aid. Participants had no contraindications for exercise (e.g. cardiac problems), and they were all following a stable steroid regime during the study period. The medical ethics committee of the VU University medical centre in Amsterdam approved the study, and all participants and their parents provided written informed consent prior to initiation of any study procedures.

### Study procedures

Participant attended three measurement sessions over the course of 1 year: a practice session to familiarize the boys with the study protocol, an initial measurement session at baseline (T1) and subsequently at the 1-year follow-up (T2). All measurements were performed at the rehabilitation outpatient clinics of the VU University Medical Center in Amsterdam, or at the Radboud University Medical Center in Nijmegen. During each assessment, a short physical examination was carried out to determine weight, height and leg length, followed by the performance of the two walking tests, the first to assess metabolic walking energy expenditure and comfortable walking distance and the second to assess maximal walking distance. A 30-minute resting period was allowed between the two walking tests.

### Measurements and instrumentation

Metabolic walking energy expenditure was assessed using the walking energy cost test (WECT), which has been described previously by Kempen et al [5]. During the WECT, subjects first sat comfortably on a chair for a 6-minute rest measurement, and then walked for 6 minutes at a self-preferred comfortable speed on an indoor oval course. Throughout the test, heart rate (HR) was recorded with a polar band (Polar RS400, Polar Electro Oy, Kempele, Finland) and pulmonary gas exchange was measured with an accurate [15], lightweight gas-analysis system (Metamax 3B, Cortex Biophysik, Leipzig, Germany) that was worn on the shoulders. Furthermore, the walked distance at comfortable speed was assessed.

The walked distance at maximal speed was assessed with the 6-minute walk test (6MWT), which was conducted according to American Thoracic Society methods [16]. The test was performed on a 25 m straight indoor course, with cones positioned at each end. Boys were instructed to walk as fast as possible from one end to the other, going around the cones, though they were allowed to stop and rest when necessary. Throughout the test, HR was recorded with a polar band.

### Data analysis

During the WECT, steady state periods were determined over the last three minutes of the rest test and the walk test. The mean speed and mean oxygen-uptake values (VO_2_, in mlkg^−1^min^−1^) and respiratory exchange ratios (RER) were computed over these steady state periods and these values were then used to calculate the average resting and walking energy consumption demands (ECSrest and ECSwalk, both in Jkg^−1^min^−1^), as defined by (4.940 * RER + 16.040) * VO_2_ [17]. To control for the influence of age, energy consumption values were normalized according to the scheme by Schwartz et al, which allows elimination of changes due to the different body dimensions of children, adolescents and adults [18]. Based on this scheme, the following parameters were calculated according to the formulas outlined in S1 Appendix: a) the walking effort, defined by the mean net non-dimensional energy consumption (NN-ECS), i.e. the amount of energy consumed per kilogram body weight per minute [19]; b) the walking economy, defined by the mean net non-dimensional energy cost (NN-EC). This value is calculated by dividing NN-ECS by speed and reflects the amount of energy consumed per kilogram body weight per distance travelled [19]; and c) the walking efficiency, i.e. the walking economy as a percentage of speed-matched control cost (SMC-EC, in %). This value reflects the walking cost relative to reference values of age-matched typically developing children measured at the same normalized walking speed, which had been previously collected in the motion analysis center of Gillette Children's Specialty Healthcare (St Paul, USA). Other outcomes derived included the comfortable walking distance covered in 6 minutes (D_WECT_, in m) and the mean HR during the last tree minutes of the test (HR_WECT_, in beatsmin^−1^). From the 6MWT, the maximal walking distance covered in 6 minutes (D_6MWT_ in m) and the mean HR (beatsmin^−1^) over the last three minutes of the test were derived (HR_6MWT_).

### Statistical analysis

Statistical analyses were performed using SPSS (version 20.0, IBM Corp, Armonk, NY, USA). The two-tailed level of significance was set at *p* = 0.05. Data of this study is annexed in S1 dataset.

Demographic variables and background variables were analyzed using descriptive statistics. Summary statistics (means and standard deviations) were calculated at baseline (T1) and at 1-year follow-up (T2) for the following outcome measures: NN-ECS, NN-EC, SMC-EC, HR_WECT_, HR_6MWT_, D_WECT_ and D_6MWT_. These data are presented for the whole group and for years of age (yoa) subgroups (<8 versus ≥8 yoa)). The age of 8 was chosen based on findings of Goemans et al., showing that 7.5–8.5 yoa appears to be the age range when DMD boys present clear deterioration in walking performance [13]. Statistical comparisons were made using a two-way repeated measures analysis of variance (ANOVA) with two within subject factors (time of measurement (T1 versus T2) and age group (<8 versus ≥8 yoa).

## Results

### Participants

Fourteen boys with DMD participated in the study. The median age of the boys at baseline was 8.0 years, with a mean of 8.2 years (range 6–12.5); median weight, height and leg length were 27.4 kg, 126.5 cm and 60 cm, respectively (Table 1). Over the course of 1 year (54 weeks on average, range 51–57), boys demonstrated a statistically significant increase in weight, height, and leg length (p≤0.001). Based on information obtained from the medical records, 1 boy was on daily corticosteroids (Deflazacort) and 12 boys were on corticosteroids for 10 days on and 10 days off (Prednisone). In one boy information about steroid use could not be traced.

**Table 1:** Subject demographics.

		Baseline	1-year follow-up
Age [years]	Mean ± SD	8.2±1.9	9.2±1.9
	Median	8.0	9.0
	Range	6.0–12.5	7.0–13.5
Weight [kg]	Mean ± SD	31.2±12.8	34.9±14.2
	Median	27.4	33.5
	Range	21–67	22–75
Height [cm]	Mean ± SD	128.6±11.7	134.6±12.0
	Median	126.5	135.5
	Range	109–154	113–158
Leg length [cm]	Mean ± SD	59±8.3	61±7.5
	Median	60	62
	Range	42–74	45–74

### One-year changes in WECT and 6MWT outcomes

Summary statistics for WECT outcomes at baseline and 1-year follow-up are presented in Table 2. Over the course of the study, a significant decrease of -28 m (-8.2%, F = 5.1, p = 0.043) was noted in the walked distance at comfortable speed (D_WECT_). Besides, walking effort (NN-ECS) and walking efficiency (SMC-EC) decreased with 6.8% (F = 1.24, p = 0.287) and 4.4% (F = 0.60, p = 0. 452), respectively. One-year changes in walking economy (NN-EC) and HR_WECT_ were both smaller than −1.2% and not statistically significant (p = 0. 940 and p = 0. 785, respectively). Changes in the walked distance at maximal speed (D_6MWT_) and the mean HR_6MWT_ were also not statistically significantly (Table 3).

**Table 2:** One-year changes in WECT outcomes.

	T1^∧^ Mean (SD)	T2^∧^ Mean (SD)	ΔT2 –T1^∧^ Mean (SD)	Factor	p-value
D_WECT_ (comfortable walking distance, in m)					
Total group (n = 14)	350 (40)	322 (36)	−28 (42.6)	Time	0.043*
<8 years (n = 6)	329 (47)	314 (33)	−15 (53)	Group	0.158
≥8 years (n = 8)	365 (28)	328 (39)	−37 (33)		
				Interaction	0.344
HR_WECT_ (heart rate, in beatsmin^−1^)					
Total group (n = 12)	133 (10)	132 (13)	−0.15 (8.4)	Time	0.785
<8 years (n = 5)	138 (4)	136 (13)	−2.6 (9.7)	Group	0.449
≥8 years (n = 7)	131 (9)	132 (12)	−2.0 (8.3)		
				Interaction	0.409
NN-ECS (walking effort)					
Total group (n = 14)	.167 (.04)	.156 (.03)	−0.011 (.04)	Time	0.287
<8 years (n = 6)	.167 (.05)	.152 (.03)	−0.015 (.05)	Group	0.851
≥8 years (n = 8)	.167 (.03)	.158 (.03)	−0.009 (.03)		
				Interaction	0.769
NN-EC (walking economy)					
Total group (n = 14)	0.41 (.08)	0.42 (.07)	0.005 (.08)	Time	0.940
<8 years (n = 6)	0.42 (.12)	0.40 (.08)	−0.020 (.09)	Group	0.686
≥8 years (n = 8)	0.41 (.04)	0.43 (.06)	0.025 (.08)		
				Interaction	0.305
SMC-EC (walking efficiency, in %)					
Total group (n = 14)	175 (30)	183 (32)	+8.1 (31.9)	Time	0.452
<8 years (n = 6)	176 (46)	173 (37)	−3.7 (31.4)	Group	0.597
≥8 years (n = 8)	174 (16)	191 (29)	+17 (31.4)		
				Interaction	0.248

Abbreviations: T1, measurement at baseline; T2, measurement at 12 months; WECT, walking energy cost test; NN-ECS, net nondimensional energy consumption; NN-EC, net nondimensional energy cost; SMC-EC, speed-matched control energy cost.

* Significant time effect at p<0.05.

**Table 3:** One-year changes in 6MWT outcomes.

	T1^∧^ Mean (SD)	T2^∧^ Mean (SD)	ΔT2 –T1^∧^ Mean (SD)	Factor	p-value
D_6MWT_ (maximal walking distance, in m)					
Total group (n = 14)	433 (60.1)	404 (59.3)	−29 (55.1)	Time	0.086
<8 years (n = 6)	413 (70.1)	415 (78.4)	1.3 (63.6)	Group	0.802
≥8 years (n = 8)	448 (51.1)	396 (44.3)	−52 (37.1)		
				Interaction	0.072
HR_6MWT_ (heart rate, in beatsmin^−1^)					
Total group (n = 11)	154 (14)	155 (15)	0.82 (11.3)	Time	0.466
<8 years (n = 4)	154 (4.6)	162 (6.1)	8.3 (2.6)	Group	0.518
≥8 years (n = 7)	154 (17)	150 (18)	−3.4 (12.3)		
				Interaction	0.099

### One-year changes according to age groups

**Figure 1:**
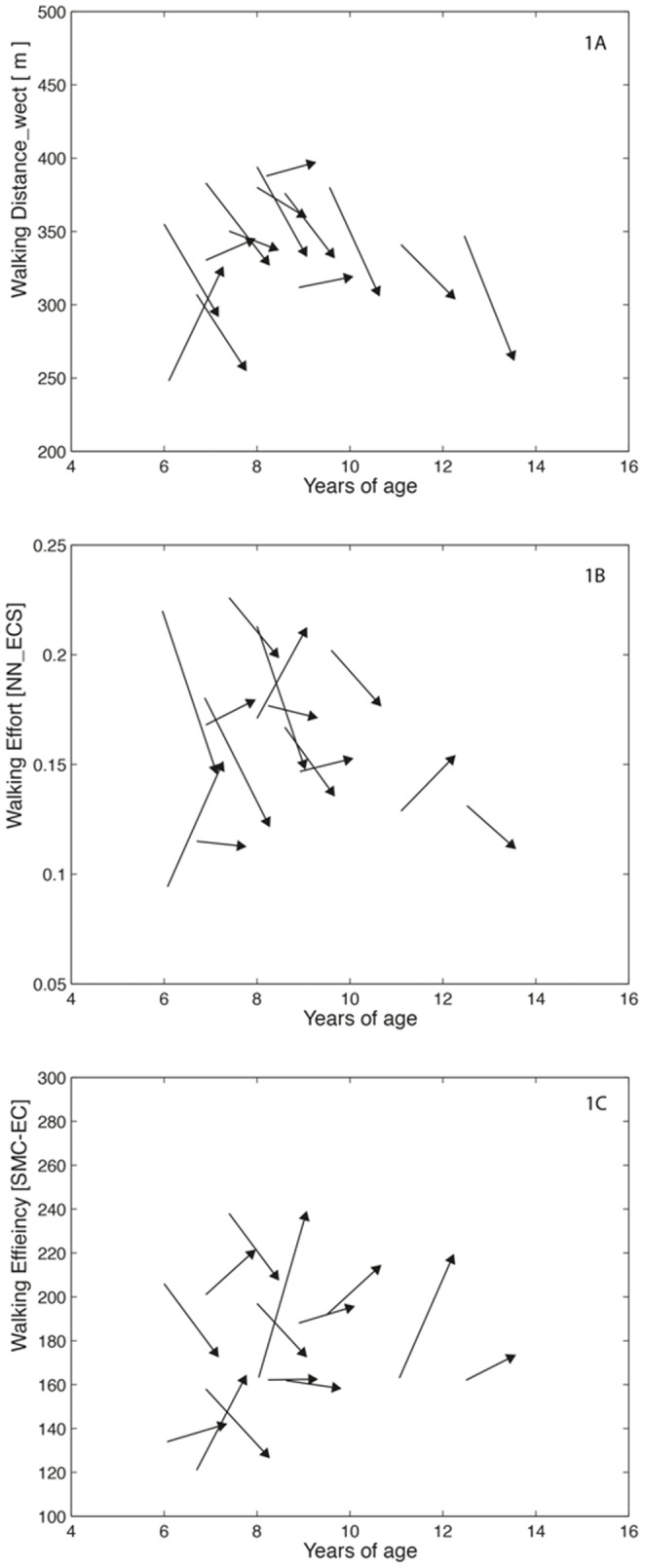
One-year change in (A) comfortable walking distance; (B) walking effort; and (C) walking efficiency, as measured with the WECT.

On the WECT, children below 8 yoa showed a smaller annual decrease in the walked distance at comfortable speed (D_WECT_) compared to children above 8 yoa (−15 m (−5%) for boys <8 yoa versus −37 m (−11%) for boys ≥8 yoa, Table 2, Fig. 1A and S1-A Figure), whereas the opposite was seen for walking effort, i.e. in boys younger than 8 yoa a larger annual decrease in NN-ECS (-9%) was noted compared to boys older than 8 yoa (−5%), Table 2, Fig. 1B and S1-B Figure. Correspondingly, in the older boys, SMC-EC increased with 17 percentage points (pp, i.e. +10% of the mean), while in the younger boys it slightly decreased (−3.5 pp (−2%); Table 2, Fig. 1C and S1-C Figure). However, the main effect of age group on each of these outcomes was not significant (p>0.158), and also there were no interaction effects (p>0.248).

**Figure 2:**
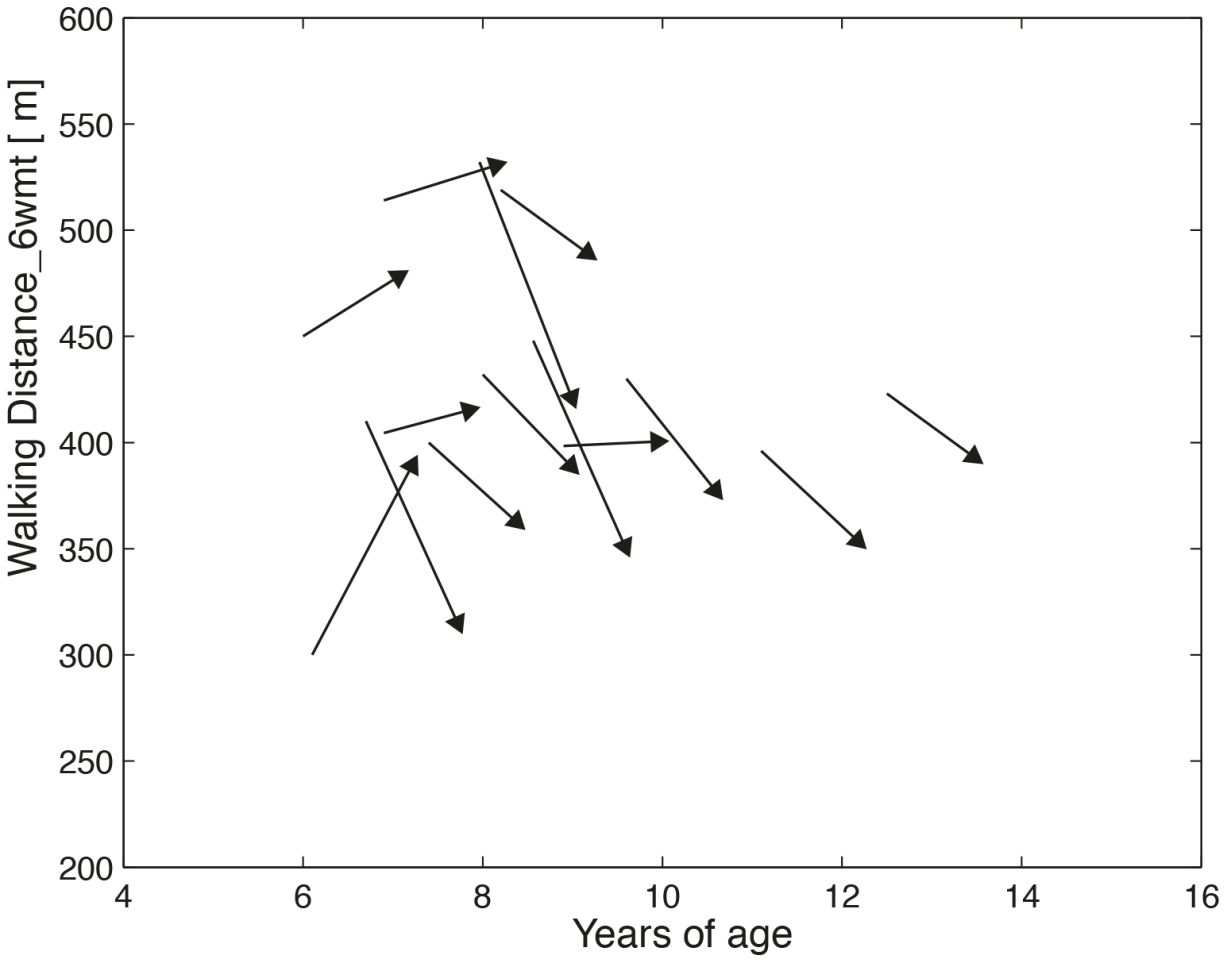
One-year change in maximal walking distance, as measured with the 6MWT.

With respect to the 6MWT, it was found that in boys below 8 yoa the walked distance at maximal speed (D_6MWT_) remained unchanged (+1.3 m (+0.3%)), while in boys above 8 yoa it significantly decreased (−52 m (−11.6%), Table 3, Fig. 2 and S1-D Figure).

## Discussion

In this study in ambulant boys with DMD, it was found that walking distance at comfortable speed decreased significantly over the course of one year with 8%, while metabolic walking energy expenditure, measured as SMC-EC, increased with 4.4%, although this change was not significant. Changes were different according to age. Boys who were below 8 yoa showed a slight improvement in walking energy expenditure (−2%), while in boys above 8 yoa, deterioration was observed (+10%).

However, the main effect of age on walking energy expenditure was not significant, and also there was no significant interaction effect.

Since this study was the first to evaluate longitudinal change in walking energy expenditure in DMD, our results could not be directly compared. A comparison with the results obtained from a cross-sectional study in 43 children with cerebral palsy showed that the 1-year increase in walking energy expenditure found in our study was much larger than that reported by Marconi and colleagues [20]. In the Marconi study, the change in locomotory index (a measure comparable to our SMC-EC outcome) between children aged 4–7 and children aged 8–11 was -1% when assessed at matching gait speed (3 km/h), which is smaller than the 4.4% increase in SMC-EC found in the present study. Likely, this divergence is due to a difference in disease characteristics of the respective participants, since DMD is portrayed by the development of severe progressive muscle weakness over time, which is not seen in cerebral palsy. As shown by Brehm et al, the extent of muscle weakness in the lower extremities is directly related to metabolic walking energy expenditure, with energy expenditure increasing with increasing muscle weakness [21].

**Figure 3:**
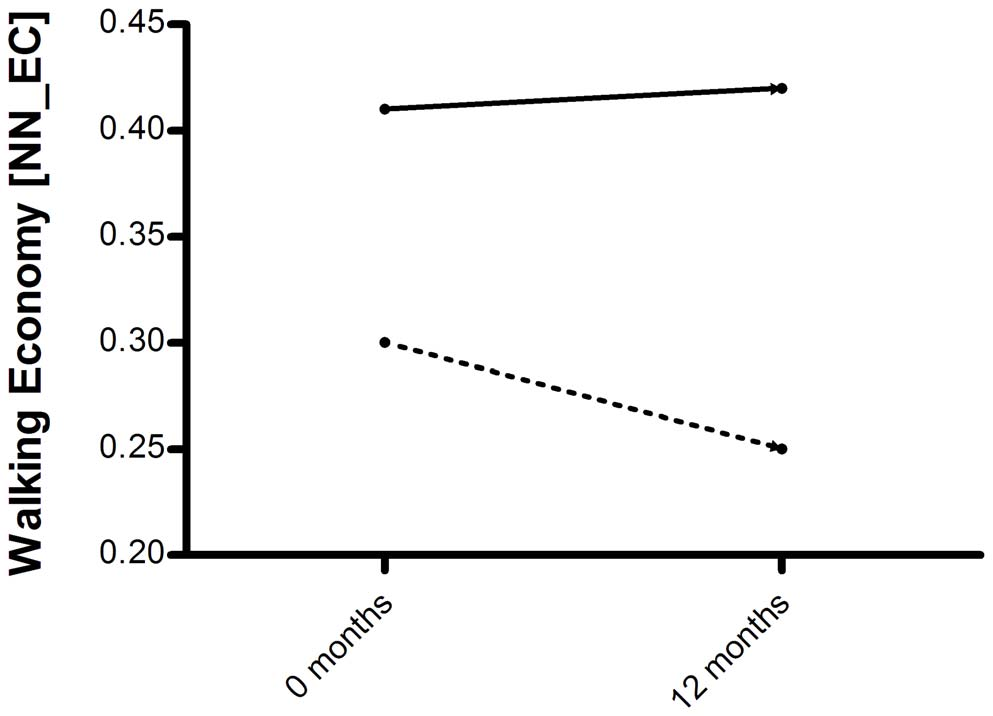
One-year change in walking cost in typically developing children (straight line) and in boys with DMD (dashed line). Reference data of typically developing children are based on Thomas et al [23].

Our observation that comfortable walking distance decreases with age, whilst energy expenditure increases is contrary to age-related trends seen in typically developing children. Typically developing children experience increases in walking distance with maturation, while energy expenditure decreases, both in terms of walking consumption [8], [22] and walking cost [8], [9], [23]. Thomas and colleagues, for example, reported a 1-year decrease in walking cost (measured as NN-EC) in typically developing children (Fig. 3), corresponding to an improvement in walking economy of about 17% [22]. In contrast, we found a 1.2% increase in NN-EC over the course of one year, indicating that walking economy slightly declined. However, it must be noted that the mean age of the children in the Thomas study was somewhat higher (11.1 years) compared to the mean age of our study sample (8.2 years), which hinders an age specific comparison with reference data of walking energy cost, as advocated in the literature [9].

The increase in walking energy cost found in our study was more apparent in older boys, who also showed the largest decrease in comfortable walking distance. In accordance with the energy minimization hypothesis [24], [25], these results suggest that boys with DMD attempt to minimize increases in walking effort by lowering their gait speed and thus maintain walking over longer distances. While in the younger boys, a slight reduction in gait speed appeared to effectively reduce the walking effort, ambulating at a sustainable effort for the older boys seems only possible at a severely reduced gait speed (Fig. 1a and Fig. 1b), subsequently raising the energy cost relative to speed-matched control cost (Fig. 1c). The raise in energy cost seen in these boys may partly be due to the increasing gait deviations that result from progressive muscle weakness secondary to muscle wasting [26]–[28]. This suggestion is underlined by findings from Thomas et al., who showed that DMD boys presenting with more severe gait deviations had the highest walking cost at the lowest comfortable speed [29]. Additional studies in larger cohorts of boys with DMD are needed to confirm our findings and to further study the age-related changes in walking cost seen in this population, in relation to concomitant changes in kinematics and kinetics of gait. This information will be useful in designing clinical trials of therapeutic interventions that aim to slow the decline in walking performance in DMD [30].

The fact that 1-year changes in walking cost were not statistically significant, may be due to our small sample size and the heterogeneity of the data, expressed by the large SD change. However, also on the 6MWT a large heterogeneity in test outcome over time was observed, a finding consistent with previous reports [10]-[14]. Nonetheless, at present, the 6MWT is regarded the most reliable clinical endpoint in DMD, requiring the smallest patient population for statistical power [31]. In that respect, the WECT may offer a promising alternative, considering the significant change in comfortable walking distance observed on this test. Besides, the WECT may offer advantages in DMD as a submaximal measure of ambulatory function, allowing a reduction in the rather high physiologic stress experienced when walking function is assessed with the 6MWT, as shown by the higher HR values. However, it is important to keep in mind the difficulty of testing boys with behavioral problems when using the WECT in research or clinical practice [5].

One of the strengths of this study is that data on the boys participating in the study represent a decline in walking performance under current standards of care in the Netherlands. The small number of boys included in the study can be considered a limitation, since this makes it difficult to draw firm conclusions regarding the change in walking energy expenditure over one year, especially with respect to age subgroups. Yet, irrespective of the small sample size, we were able to collect some information that may be useful for future clinical trials investing therapeutic interventions aimed at reducing walking energy expenditure.

## Conclusion

The results of our study show that metabolic energy expenditure can be measured longitudinally in DMD and provides extra information on the natural history, next to walking distance. Data suggest that the natural course of walking performance in ambulant boys with DMD is characterized by a decrease in walking distance at comfortable speed and an increase in walking energy cost. The rate of energy cost seems to increase with age, while walking distance decreases, which is opposite to the trend observed in typically developing children, as reported in the literature.

However, given our limited sample size, we cannot draw firm conclusions regarding the longitudinal change in metabolic walking energy cost in DMD in relation to age.

## Supporting Information

S1 Appendix Formulas used for the calculation of metabolic energy expenditure parameters. Based on Schwartz et al. [18].
(DOC)

S1 Dataset Dataset used for statistical analysis.
(SAV)

S1 Figure Years of age at baseline by one-year change in (A) comfortable walking distance; (B) walking effort; (C) walking efficiency, and (D) maximal walking distance.
(TIF)
