# Supplementary material for: Age-Related Longitudinal Changes in Metabolic Energy Expenditure during Walking in Boys with Duchenne Muscular Dystrophy
Source: PLoS One. 2014 Dec 15;9(12):e115200. doi: 10.1371/journal.pone.0115200 (PMC4266650; doi:10.1371/journal.pone.0115200)
Supplement: S1 Appendix — Formulas used for the calculation of metabolic energy expenditure parameters. Based on Schwartz et al. [18]. (DOC) [file pone.0115200.s001.doc]

Appendix 1. Formulas used for the calculation of metabolic energy expenditure parameters. Based on Schwartz et al. [18]

**Walking effort**

Where m = body weight (kg) and g = 9.81

**Walking economy**

Where v = speed (ms^-1^); m = body weight (kg) and g = 9.81

**Walking efficiency**

Where ;

and g = 9.81
